# A DR6/p75^NTR^ complex is responsible for *β*-amyloid-induced cortical neuron death

**DOI:** 10.1038/cddis.2013.110

**Published:** 2013-04-04

**Authors:** Y Hu, X Lee, Z Shao, D Apicco, G Huang, B J Gong, R B Pepinsky, S Mi

**Affiliations:** 1 Department of Discovery Neurobiology, BiogenIdec Inc., 14 Cambridge Center, Cambridge, MA, USA

**Keywords:** DR6, p75^NTR^, A*β*, AD, neurodegenerative disease, neuronal cell death

## Abstract

The p75 neurotrophin receptor (p75^NTR^) is a known mediator of *β*-amyloid (A*β*)-induced neurotoxicity implicated in Alzheimer's disease (AD). Here, we demonstrate that death receptor 6 (DR6) binds to p75^NTR^ and is a component of the p75^NTR^ signaling complex responsible for A*β*-induced cortical neuron death. Cortical neurons isolated from either DR6 or p75^NTR^ null mice are resistant to A*β*-induced neurotoxicity. Blocking DR6 function in cortical neurons by anti-DR6 antibodies that block the binding of DR6 to p75^NTR^ receptor complex or by a dominant negative DR6 construct lacking the cytoplasmic signaling death domain attenuates A*β*-induced caspase 3 activation and cell death. DR6 expression is upregulated in AD cortex and correlates with elevated neuronal death. Targeting the disruption of the DR6/p75^NTR^ complex to prevent A*β* cytotoxicity represents a new approach for the treatment of neurodegenerative disorders such as AD.

Alzheimer's disease (AD) is a neurodegenerative disorder characterized by progressive cell death and subsequent cognitive impairment including memory loss. An early and constant feature of AD is the degeneration of cholinergic neurons in the basal forebrain that innervate the hippocampus and neocortex.^1^ Although the cause of disease is unclear, the deposition of *β*-amyloid (A*β*) into amyloid plaques is the hallmark pathological feature of AD. Many of the new experimental therapies for AD attempt to prevent the formation, aggregation, and accumulation or the facilitation of A*β* removal from preexisting deposits.

Tumor necrosis factor receptor (TNFR) superfamily members, which have roles in immunological and oncological diseases,^2, 3^ have recently emerged as drug targets for the treatment of neurodegenerative diseases such as Alzheimer's and central nervous system (CNS) demyelination diseases.^4, 5, 6^ A subset of the TNFR superfamily, the death receptors, contains four highly conserved cysteine-rich regions in their ectodomain and a cytoplasmic death domain that upon receptor oligomerization activates diverse downstream targets, including caspases.^7, 8^ Eight death receptors have been identified and each regulates cell death in selected cell populations:^8, 9^ Fas (also known as death receptor 2, CD95, and APO-1), TNFR1 (tumor necrosis factor receptor 1), TRAMP (also known as death receptor 3), TRAILR1 (TNF-related apoptosis-inducing ligand receptor 1), TRAILR2, DR6 (death receptor 6), ectodermal dysplasia receptor, and p75^NTR^ (p75 neurotrophin receptor). Fas-induced cell death has a critical immunomodulatory role in the killing of autoaggressive lymphocytes and pathogen-infected cells.^10^ TRAILRs have a critical role in apoptosis of tumor cells.^2^ In the CNS, p75^NTR^ has a well-established role in neuronal cell death and axon degeneration. p75^NTR^ forms a receptor complex with sortilin that binds pro-nerve growth factor to induce neuronal cell death.^6, 11^ p75^NTR^ also forms a tripartite complex with NogoR (Nogo receptor) and LINGO-1 (Leucine-rich repeat and Ig domain containing NogoR interacting protein 1) to inhibit axon outgrowth.^12^ In addition, p75^NTR^ has been shown to bind A*β* to induce cell death in hippocampal neurons *in vitro* and cholinergic basal forebrain neurons *in vivo.*^13, 14^

There is increasing evidence that DR6 has an important role in neuronal cell death. Nikolaev *et al.*^4^ have reported that DR6 induces neuronal cell death and axon degeneration during CNS development by directly binding N-terminal A*β* precursor protein in the absence of trophic factors through activation of the caspase 6 and casp6 signaling pathway.^4^ DR6 also mediates oligodendrocyte cell death during development.^5^ Here, we demonstrate that DR6 forms a receptor complex with p75^NTR^ to induce cortical neuron death. Anti-DR6 antibody that blocks the formation of the DR6/p75^NTR^ receptor complex significantly reduces A*β*-induced neurotoxicity. DR6 and p75^NTR^ expression levels are upregulated in AD brains. Blocking DR6/p75^NTR^ receptor complex function represents a novel therapeutic target for the treatment of neurodegenerative diseases such as AD.

## Results

### DR6 expression levels are correlated with neuronal death

**Figure 1:**
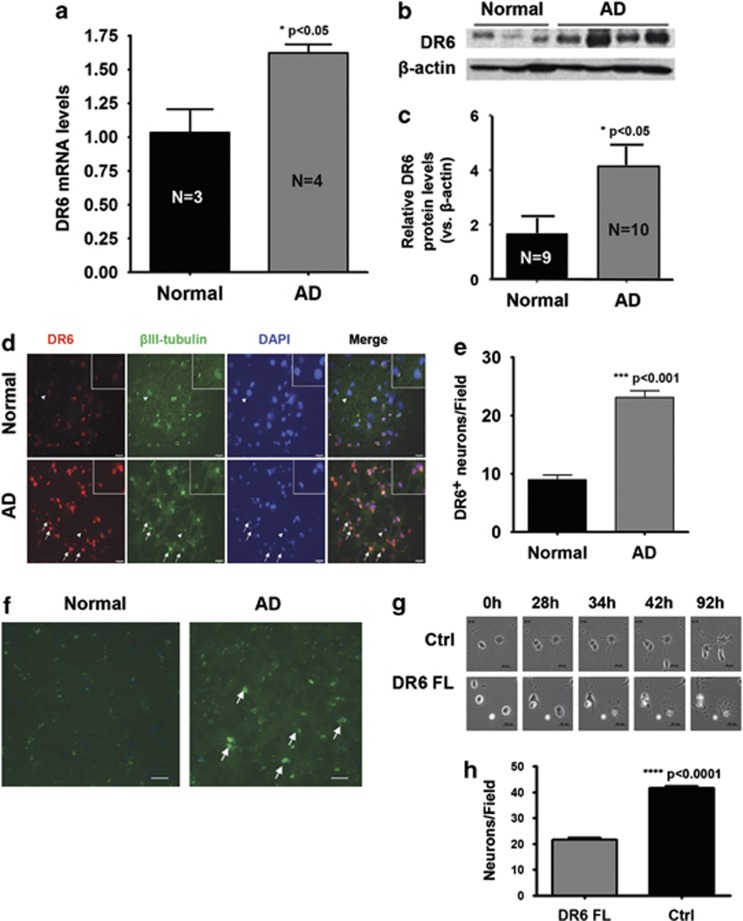
DR6 is expressed in cortical neurons and upregulated in AD. (**a**) Quantitative RT-PCR analysis of DR6 mRNA expression in AD. (**b**) Western blot analysis of DR6 expression from four AD and three age-matched normal brains. (**c**) Densitometry quantification of DR6 protein level from DR6 western blotting from 10 AD and 9 age-matched normal brains. (**d**) *In-situ* hybridization analysis of DR6 (red) and co-localization with neurons (*β*III-tubulin, green) in normal and AD cortex (yellow=merge, blue=DAPI). Arrows indicate cells with nuclear condensation characteristic of apoptosis. Arrowheads indicate area of image enlargement (top right corner). Scale bar=25 *μ*m. (**e**) Quantification of DR6^+^ neurons from **d.** (**f**) Immunocytochemical staining of DR6 from age-matched normal and AD brains; arrows indicate intensely stained DR6-positive cells. Scale bar=95 *μ*m. (**g**) Time-lapse images showing the effect of full-length DR6 (DR6 FL) overexpression in cultured neurons 0–92 h after transfection (bottom panel) versus vector control-transfected neurons (top panel). Scale bar=20 *μ*m. (**h**) Quantification of neuronal survival from **g**

DR6 is expressed in cortical neurons. To determine whether DR6 contributes to AD pathology, mRNA and protein expression levels were evaluated using post-mortem brain samples of AD patients with age-matched normal controls. Quantitative real-time PCR (RT-PCR) and western blot results (Figures 1a–c) showed a 1.6-fold and a 2.5-fold increase in DR6 mRNA and protein levels, respectively, in AD brain samples compared with normal brain samples. Consistent with this finding, *in-situ* hybridization revealed a 2.5-fold increase in the number of DR6-positive (DR6^+^) neurons in the cortex of AD brains compared with normal human brains (Figures 1d and e). Cells that displayed nuclear DNA condensation characteristic of apoptosis (Figure 1d, arrows) showed increased DR6 staining (red) when compared with normal brain cells (Figure 1d), suggesting that upregulation of DR6 may contribute to neuronal cell death. Immunocytochemical staining using anti-DR6 antibody also demonstrated an increased number of DR6-positive neurons with more intense staining in the AD brains compared with age-matched normal brain tissue (Figure 1f).

To further confirm that DR6 expression level contributes to neuronal death, full-length DR6 (DR6 FL) was introduced into neocortical neurons by lentivirus infection. Time-lapse live images captured across 92 h revealed that ectopic expression of DR6 FL-induced neuronal death as evident by changes in cell morphology and a decrease in cell count (Figures 1g and h). DR6 FL-infected neurons showed a twofold reduction in cell survival compared with control virus-infected neurons (Figure 1h). The increased expression of DR6 in AD brains and increased number of apoptotic cells in cultured neocortical neurons overexpressing DR6 FL suggest an important role for DR6 in neuronal cell death.

### DR6 and p75^NTR^ form a receptor complex

**Figure 2:**
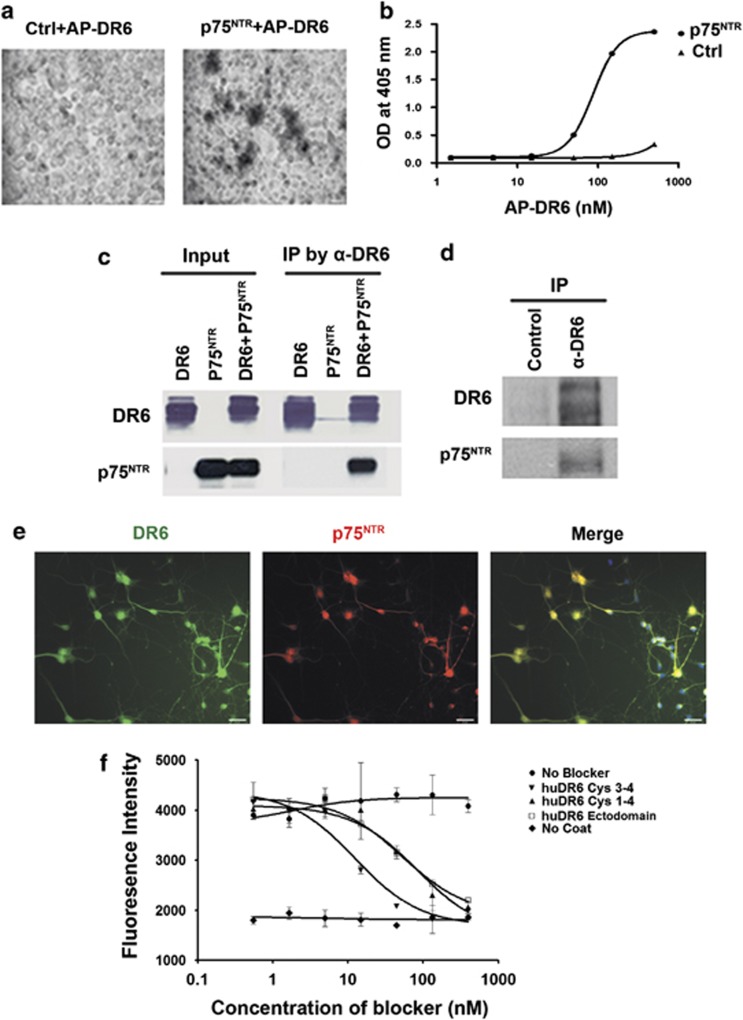
DR6 and p75^NTR^ form a receptor complex. (**a**) AP-DR6 binding to HEK 293 cells expressing p75^NTR^ and not to untransfected control cells. The non-homogenous staining pattern reflects incomplete transfection. Scale bar=25 *μ*m. (**b**) Quantitative assessment of **a** by ELISA. (**c**) DR6/p75^NTR^ co-immunoprecipitation in HEK 293 cells overexpressing Myc-tagged DR6 and p75^NTR^. (**d**) DR6/p75^NTR^ co-immunoprecipitation in fetal human spinal cord lysates. (**e**) Immunocytochemical staining to co-localized the DR6 and p75^NTR^ expression in cultured neurons. Scale bar=25 *μ*m. (**f**) Competition ELISA showing the binding of europium-labeled human p75^NTR^ to human DR6 ectodomain-coated plates in the presence and absence of blocker constructs

The upregulation of DR6 expression in AD brain tissues suggests that DR6 may contribute to neurodegeneration. This result prompted us to look for a ligand or co-receptor, which participates with DR6 to induce cortical neuron death. As p75^NTR^ also contains a death domain and is also upregulated in AD cortical and hippocampal neurons,^15, 16^ we investigated whether DR6 binds to p75^NTR^. First, we tested whether alkaline phosphatase–DR6 fusion protein (AP-DR6) could bind HEK 293 cells expressing p75^NTR^. As shown in Figure 2a, AP-DR6 bound strongly to cells expressing p75^NTR^ compared with control non-transfected cells with an EC_50_ of 90 nM (Figure 2b). Second, to determine whether DR6 forms a receptor complex with p75^NTR^, DR6 was immunoprecipitated from HEK293 cells co-transfected with p75^NTR^ and Myc-tagged DR6. The presence of p75^NTR^ in the immunoprecipitate was analyzed by anti-p75 western blot (Figure 2c). In the input lanes, both p75^NTR^ and DR6 expressions were detected in transfected cells; however, a strong p75^NTR^ immunoreactive band was only detected in the DR6/p75^NTR^ co-transfected precipitate and no band was detected in the cells transfected with either DR6 or p75^NTR^ alone. Third, we determined if the DR6/p75^NTR^ receptor complex is present endogenously by subjecting human fetal spinal cord lysates to immunoprecipitation with an anti-DR6 antibody followed by western blot analysis to determine if p75^NTR^ co-precipitates with DR6. As shown in Figure 2d, DR6 and p75^NTR^ immunoreactive bands were detected. Fourth, an immunocytochemistry (ICC) study was performed to confirm the co-expression of DR6 and p75^NTR^ in aged cultured cortical neurons (Figure 2e), and greater than 85% of cultured neurons were DR6 and p75^NTR^ double positive. The above four studies indicate that DR6 and p75^NTR^ form a receptor complex.

We next determined which region of DR6 ectodomain binds to p75^NTR^. Deletion constructs lacking different cysteine-rich domains (CRDs) of DR6 were generated and co-expressed with p75^NTR^ in HEK 293 cells. p75^NTR^ co-immunoprecipitated with DR6 FL protein and deletion mutants lacking CRD 1 and 2, but not DR6 protein lacking CRD 3 or CRD 4, or CRD 3-4 double deletion (data not shown), suggesting that p75^NTR^ binds to CRD 3-4 of the DR6 ectodomain. To verify this finding, we directly measured the binding of recombinant DR6 to p75^NTR^ in a competition ELISA (Figure 2f). DR6 full ectodomain, CRD 1-4, and CRD 3-4 all bound to p75^NTR^ with an IC_50_ of ≤30 nM.

### Neurons from DR6 null mice are resistant to A*β*-induced cell death

**Figure 3:**
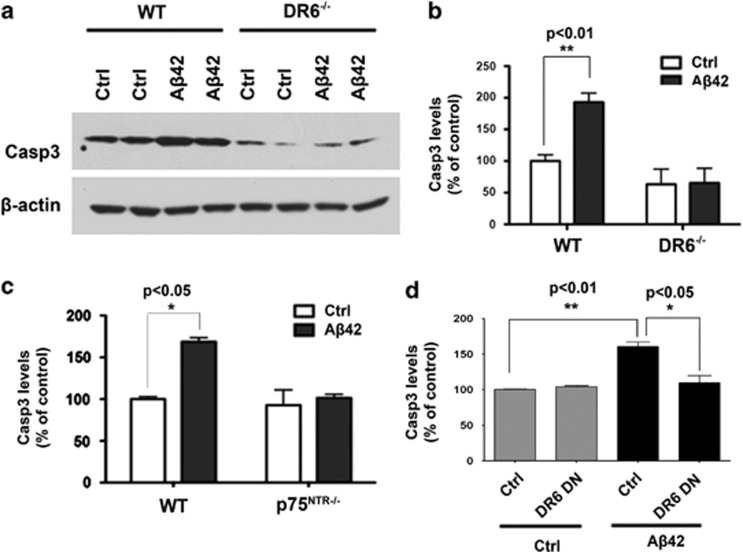
Cortical neurons isolated from DR6 and p75^NTR^ null mice are resistant to A*β*42-induced cell death. (**a**) Western blot analysis of cleaved casp3 levels in DR6^−/−^ cortical neurons after A*β*42 exposure. (**b**) Quantification of cleaved casp3 levels from **a**. (**c**) Quantification of cleaved casp3 levels by western blot analysis in p75^NTR−/−^ cortical neurons after A*β*42 exposure. (**d**) Quantification of cleaved casp3 levels by western blot analysis in DR6-DN-infected neurons after A*β*42 exposure

To determine the contribution of both DR6 and p75^NTR^ to A*β*-induced neurotoxicity, cortical neurons isolated from DR6 and p75^NTR^ null mice (E16) were cultured for 4 days, and then treated with aggregated A*β*42 for 24 h. Cell death was monitored by measuring the expression levels of cleaved caspase 3 (casp3) by western blot (Figure 3). When compared with control treated cells, A*β*42 treatment induced a twofold increase in cleaved casp3 expression in wild-type neuron cultures, but had no effect on neurons isolated from DR6 null mice (Figures 3a and b). Consistent with the DR6 null neuron casp3 data, there was a twofold increase in casp3 activation in wild-type neurons treated with A*β*42, with no differences observed in p75^NTR^ null cortical neurons (Figure 3c). Thus, although we were able to confirm that p75^NTR^ is necessary for A*β*-induced neurotoxicity in the p75^NTR−/−^ neurons, the lack of an effect of neurotoxicity in the DR6^−/−^ neurons implies that both p75^NTR^ and DR6 contribute to the A*β*42-induced cytotoxicity, a role that has long been attributed to p75^NTR^ alone.^13, 14^

To further elucidate the contribution of DR6 to A*β*42-induced cytotoxicity, a dominant negative version of DR6 lacking the cytoplasmic death domain (DR6-DN) was expressed in cultured cortical neurons by lentivirus infection and then treated with aggregated A*β*42. The cleaved casp3 levels were analyzed again by western blot (Figure 3d). A*β*42 treatment significantly increased levels of cleaved casp3 in control-infected cells, with no changes seen in DR6-DN-expressing cell cultures when compared with control infected or the absence of A*β*42 treatment. These results show that the DR6/p75^NTR^ complex responsible for A*β*-induced cortical neuron death requires the DR6 death domain.

### Antibodies that block the formation of the DR6/p75^NTR^ receptor complex inhibit A*β*-induced neuronal death

**Figure 4:**
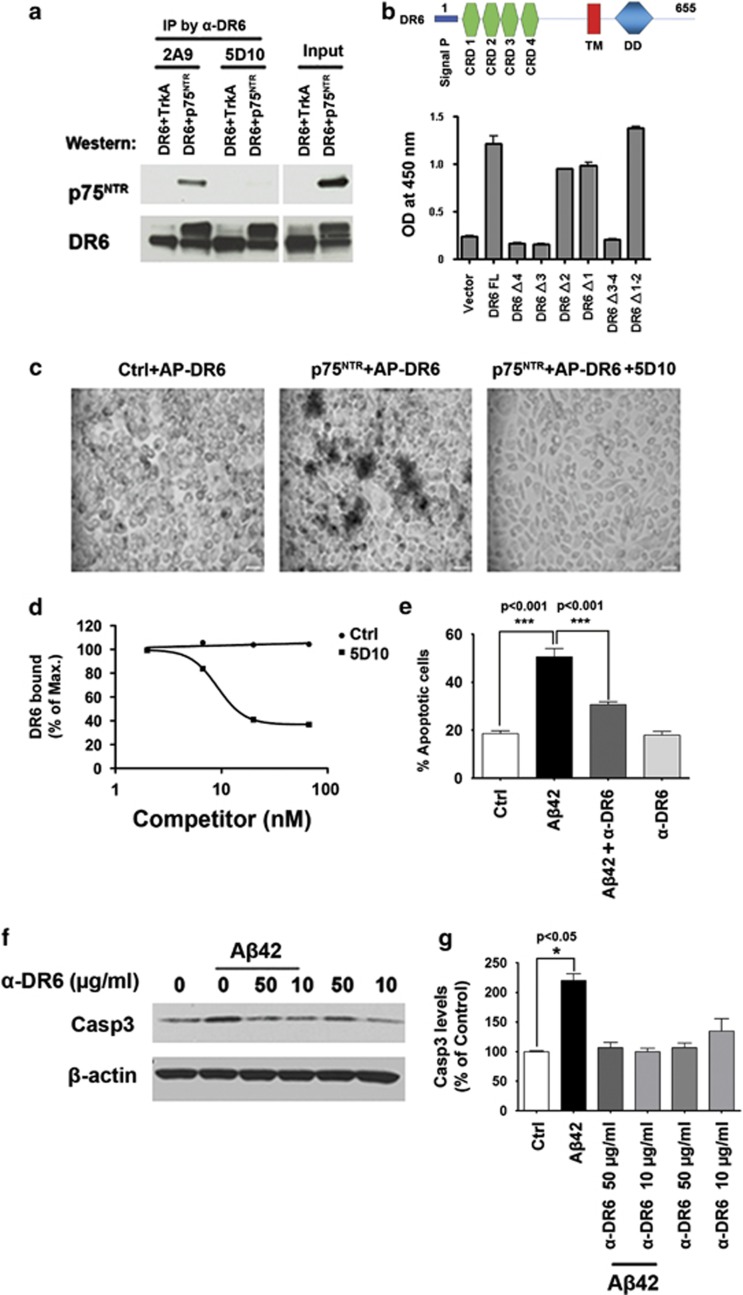
Anti-DR6 antibodies that block the interaction between DR6 and p75^NTR^ inhibit A*β*42-induced neuronal cell death. (**a**) Anti-DR6 antibody 5D10 blocked DR6 and p75^NTR^ complex formation from DR6/p75^NTR^ co-transfected HEK 293 cells. Anti-DR6 antibody 2A9, which does not bind to DR6 at the p75^NTR^-binding site, did not block p75^NTR^ from co-immunoprecipitating with DR6. (**b**) Top: Schematic domain structure of human DR6 protein depicting four extracellular CRD 1-4, a transmembrane domain (TM), and a cytoplasmic death domain (DD). Bottom: ELISA mapping 5D10-binding epitope within cysteine-rich domains of DR6 protein. (**c**) 5D10 blocks AP-DR6 binding to HEK 293 cells expressing p75^NTR^. Scale bar=25 *μ*m. (**d**) Quantitative assessment of 5D10 blocking AP-DR6 binding to p75^NTR^ from **c**. (**e**) TUNEL staining quantification of percent apoptotic cells from anti-DR6 antibody-treated and control-treated cortical neurons following A*β*42 exposure. (**f**) Western blot analysis of cleaved casp3 levels in anti-DR6 antibody-treated and control-treated cortical neurons following A*β*42 exposure. (**g**) Quantification of cleaved casp3 levels from **f**

To further confirm that the DR6/p75^NTR^ receptor complex is responsible for A*β*-induced neurotoxicity, we first assessed if anti-DR6 monoclonal antibody 5D10 affected the ability of DR6 to bind to p75^NTR^. As shown in Figure 4a 5D10 blocks the binding of DR6 to p75^NTR^ as determined by anti-DR6 immunoprecipitation and then anti-p75^NTR^ western blot in DR6/p75^NTR^ co-transfected cells. Epitope mapping revealed that the 5D10 bound within DR6 CRD 3-4 (Figure 4b). In contrast, 2A9, an antibody that binds to DR6 CRD 1-2, did not block the formation of the DR6/p75^NTR^ receptor complex (Figure 4a). In a direct binding assay, 5D10 also blocked the binding of AP-DR6 to HEK 293 cells expressing p75^NTR^ (Figure 4c). The IC_50_ for 5D10 was 9 nM (Figure 4d). Thus, 5D10 competes with p75^NTR^ by binding to the same region of DR6, thereby inhibiting the formation of the DR6/p75^NTR^ complex.

To test if 5D10 protects against A*β*42-induced neurotoxicity, cultured cortical neurons were treated with 5D10 or control antibody following aggregated A*β*42 exposure for 24 h. 5D10 treatment resulted in a twofold reduction in A*β*42-induced neuronal apoptosis by TUNEL staining (Figure 4e) and a significant decrease in casp3 activation by western blot (Figures 4f and g). Thus, 5D10 specifically binds to DR6 within the CRD 3-4 domains to block formation of the DR6/p75^NTR^ receptor complex, thereby preventing A*β*42-induced cell death in cultured neocortical neurons.

## Discussion

DR6 has emerged as an important regulator of neuronal cell death. Here, we show that DR6 is expressed in adult cortical neurons and is upregulated in the AD brain. In AD cortex, increased DR6 expression is co-localized with cells undergoing DNA condensation. To understand the linkage between DR6 overexpression and cell death, we performed an extensive series of mechanism of action studies. Most significantly, we discovered that DR6 forms a receptor complex with p75^NTR^ that mediates A*β*-induced neurotoxicity in cortical neurons. Whereas the role of p75^NTR^ in A*β*-induced cell death is well documented, our studies reveal that DR6 is essential for p75^NTR^/A*β*-induced neurotoxicity, as cortical neurons genetically deficient of either receptor were resistant to cell death induced by A*β*42 exposure. Anti-DR6 antibody that blocks the formation of the DR6/p75^NTR^ receptor complex attenuated A*β*-induced cell death. The ability of overexpression of DR6-DN to attenuate A*β*-induced cell death suggests that the death domain of DR6 is responsible for the apoptotic signal transduction. The discovery that DR6 binds p75^NTR^ and is directly involved in A*β*-induced cortical neuron death identifies a new therapeutic target for the treatment of AD.

**Figure 5:**
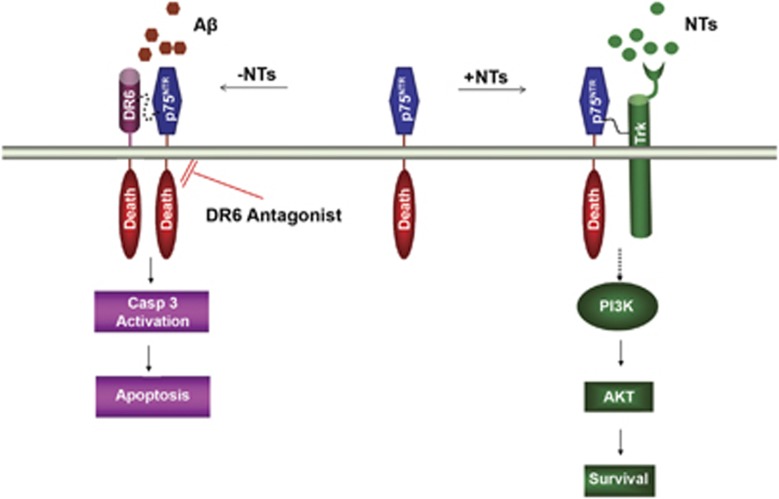
Working model for DR6/p75^NTR^ receptor complex signaling in cortical neurons. In the presence of NTs, p75^NTR^ binds NTs and Trk to promote cortical neuron survival via the AKT (also known as Protein Kinase B) pathway. In the absence of NTs, p75^NTR^ binds A*β* and DR6 to form a receptor complex, which activates the caspase 3 apoptotic signaling pathway via a cytoplasmic death domain oligomeric complex. In Alzheimer's disease, various factors might switch p75^NTR^ signaling from pro-survival to pro-death, including the increased level of A*β*, upregulation of DR6 and p75^NTR^, and decreased expression of Trk

p75^NTR^ has a complex role in regulating neuronal survival and death that is dependent on its binding to ligands and co-receptors.^6, 12, 15, 16, 17, 18, 19, 20^ p75^NTR^ is a low affinity neurotrophin (NT) receptor that interacts with Trk (tropomyosin receptor kinase) to enhance the Trk response to NTs, thereby promoting cell survival.^21^ As a cell death receptor, p75^NTR^ forms a receptor complex with sortilin to mediate pro-apoptotic signaling in response to pro-nerve growth factor binding.^11^ As a co-receptor involved in axon degeneration, p75^NTR^ forms a tripartite complex with NogoR and LINGO-1 that results in the inhibition of axon growth in response to Nogo, myelin-associated glycoprotein, or oligodendrocyte myelin glycoprotein.^12^ Several lines of evidence suggest that changes in the expression levels of p75^NTR^, its ligands, or its co-receptors during disease may switch p75^NTR^ function from pro-survival to pro-death. A*β* is increased in AD and has been shown to bind p75^NTR^ to induce neuronal death.^13, 14^ NTs, which also bind p75^NTR^ and protect neurons from A*β*-induced neurotoxicity *in vitro*,^22^ are decreased in AD.^23^ Additionally, increased p75^NTR^ and decreased TrkA expression have been reported in AD.^15, 16, 24, 25^ Our data combined with the published studies demonstrate that DR6, p75^NTR^, and A*β* are all upregulated in AD brains, whereas NTs and TrkA are downregulated, suggesting a potential role for the DR6/p75^NTR^ receptor complex in neuronal cell death in AD. Figure 5 shows a schematic model depicting the role of p75^NTR^ in regulating cortical neuron survival and death signaling. In the presence of NTs, p75^NTR^ binds NTs and Trk to promote neuronal survival. In the absence of NTs, p75^NTR^ binds A*β* and DR6 to form a death domain oligomeric complex that activates casp3 to induce cortical neuron death. DR6 antagonists selectively block the pro-apoptotic function of the DR6/p75^NTR^ complex while preserving the pro-survival function of the p75^NTR^/Trk complex. Pharmaceutical reagents capable of blocking the formation of the DR6/p75^NTR^ receptor complex, such as 5D10, could alleviate or reverse the progression of AD and other neurodegenerative diseases by promoting neuron survival.

In summary, we have discovered that DR6 and p75^NTR^ form a receptor complex that induces neuronal death upon binding of A*β*42. Studies using cortical neurons isolated from p75^NTR^ and DR6 null mice reveal that both p75^NTR^ and DR6 contribute to A*β*-induced neurotoxicity as loss of either death receptor prevented the cell death. Blocking the interaction between DR6 and p75^NTR^ represents a promising therapeutic target for AD.

## Materials and methods

### *In-situ* hybridization

Rat brain frozen sections were prepared and processed as described^26^ and were probed with digoxigenin-labeled DR6 antisense probe (5′-TAATACGACTCACTATAGGGGCTGGTGGGTAAGTTGTGGT-3′) and sense RNA probe (5′-ATTTAGGTGACACTATAGAACTCGCGGTACCTTCTCTGAC-3′). Sections were stained using the TSA plus fluorescent anti-digoxigenin conjugated antibodies kit (Perkin Elmer, Waltham, MA, USA) following the manufacturer's instructions. Sections were co-stained with anti-*β*III tubulin antibody (EMD Millipore, Billerica, MA, USA).

### RT-PCR

Quantitative RT-PCR was performed using six snap-frozen brain tissue blocks from four different AD individuals (four frontal lobes, one temporal lobe, one basal ganglion and four unspecified regions collected from four individual donors, two females, two males; Cooperative Human Tissue Network, Philadelphia, PA, USA). Three normal matching brain blocks from three individuals (two females and one male) without neurological diseases were included as control. All mRNAs were extracted using the Absolutely RNA miniprep kit (Agilent Technologies, Inc, Santa Clara, CA, USA) following the manufacturer's instruction. cDNAs were generated using purified RNAs (High Capacity cDNA Archive Kit, Life Technologies, Grand Island, NY, USA) and served as the template for quantitative RT-PCR. TaqMan Gene Expression system (Mx3000P) was used to quantify the DR6 mRNA levels using premixed primer sets with MGB probes: DR6 (Mm00446361_m1, Life Technologies). *β*-Actin mRNA levels were quantified in parallel for normalization using primer/probe set Rn00667869_m1 (Life Technologies).

### Cell culture

Rat neocortical neurons were prepared from E18 Sprague–Dawley rats (Charles River, Wilmington, MA, USA). Mouse cortical neurons were prepared from E16 DR6 null, p75^NTR^ null or WT (C57BL/6) mice. Briefly, cerebral cortices from the rat or mouse embryos were dissected out, minced, and incubated in 0.25% Trypsin-EDTA (Life Technologies) at 37 °C for 10 min. The cells were triturated after adding 10 *μ*g/ml DNase I (Sigma, St. Louis, MO, USA) and 10% fetal bovine serum (Life Technologies) to stop the reaction. Cell pellets collected by centrifugation at 275 r.c.f. at room temperature for 5 min were mechanically dissociated by gently passing through a plastic pipette until no large fragments were visible. Tissue culture plates (Corning, Corning, NY, USA) were coated with 100 *μ*g/ml Poly-𝒟-lysine (Sigma) before cell seeding. Plating densities of 1 × 10^6^/well on a 12-well plate for western blots and 2 × 10^4^/well of 4-well chamber slides (LabTek, Fisher Scientific, Pittsburgh, PA, USA) for ICC and TUNEL assay were used. Cells were maintained in Neurobasal medium containing B27 supplement (Life Technologies) at 37 °C in humidified air with 5% CO_2_ and allowed to mature 5 days before treatment.

### DR6 FL and DR6-DN plasmid construction and cell infection

Full-length human DR6 (DR6 FL; amino acid residues 1-655) DNA sequence was inserted into the NotI sites of HRST-IRESeGFP lentivirus vector (gift from R Mulligan, Harvard University) using oligonucleotide primers 5′-CACGGGATCCGCGGCCGCATGGGGACCTCTCCGAGCAGC-3′ and 5′-CAGGGATCCGCGGCCGCCTACAGCAGGTCAGGAAGATGGC-3′. Dominant-negative mouse DR6 (DR6-DN; amino-acid residues 1–370) DNA sequence was inserted into the Not I sites of HRST-IRESeGFP lentivirus vector using oligonucleotide primers 5′-CATAGTGCGGCCGCATGGGGACCCGGGCAAGCAGCATCACC-3′ and 5′-CCAGATGCGGCCGCCTAGATACTGCACACCACTATCAGCACCAGG-3′. Single and double CRD deletion DR6 constructs were synthesized from DR6 FL by site-directed mutagenesis. DR6-DN and GFP control plasmids were transfected into HEK 293 cells to produce lentivirus as previously described.^27^ Neocortical neurons were infected with lentivirus at a multiplicity of infection of one per cell.

### Time-lapse analysis

Rat cortical neurons were subjected to time-lapse recording for 92 h after cells were infected with DR6 FL or control lentivirus. Neurons were cultured in neurobasal medium with B27 supplement on a poly-ℒ-lysine-coated 24-well tissue culture plate. The entire plate was placed onto a motorized stage (Zeiss, Thornwood, NY, USA) that was housed inside a sealed chamber (Zeiss) to maintain the culture at 37 °C in humidified air with 5% CO_2_ during the entire recording. Six wide-field microscopic images of cells under each treatment were acquired through the transmitted light channel on a Zeiss Axiovert microscope equipped with a digital camera and the Axiovision software (Zeiss).

### A*β* preparation

A*β*42 peptide was obtained from Life technologies and reconstituted according to the manufacturer's instruction. First, the peptide was dissolved in HPLC grade water at 6 mg/ml, then further diluted in Dulbecco's phosphate-buffered saline (PBS) to the final concentration of 1 mg/ml and incubated at 37 °C for 24 h for aggregate formation. A measure of 50 *μ*g/ml of A*β*42 was used for all the experiments. Reverse sequence peptide A*β*(42-1) prepared in the same way was used as controls.

### Western blots

Cortical neurons were lysed in 120 μl lysis buffer (50 mM HEPES, pH 7.5, 150 mM NaCl, 1.5 mM MgCl_2_, 1 mℳ EGTA, 1% Triton X-100, and 10% glycerol) in the presence of protease inhibitor cocktail (Roche, Indianapolis, IN, USA) at 4 °C. After centrifugation at 375 r.c.f. for 15 min, the supernatants were boiled for 5–10 min in Laemmli sample buffer, subjected to 4–20% SDS-PAGE, and analyzed by western blotting with rabbit anti-cleaved capase-3 (1:1000, Cell Signaling, Danvers, MA, USA) and rabbit anti-*β*-actin (1:5000, Sigma) antibodies. Primary antibodies were visualized using anti-rabbit IgG-horseradish peroxidase (1:5000, Bio-Rad, Hercules, CA, USA). Band intensities were quantified by densitometry. AD brain tissues (five females and five males) and normal controls (five females and four males) were lysed in RIPA buffer (50 mM Tris, pH 7.2, 1% Triton X-100, 0.5% sodium deoxycholate, 0.1% SDS, 150 mM NaCl, 10 mM MgCl_2_, 5% glycerol) at 1 ml buffer/0.2 g of tissue at 4 °C overnight, followed by 15 min centrifugation at 375 r.c.f. A measure of 30 *μ*g proteins from the supernatant were subjected to western blotting analysis. For the analysis of AD samples, 10 snap-frozen brain tissue blocks from 10 different AD individuals (four frontal lobes, six hippocampus purchased from Tissue Solutions, Glasgow, UK). Matching brain blocks from nine normal individuals (three frontal lobes, six hippocampus purchased from Tissue Solutions). DR6 expression was detected using anti-DR6 antibody (6A12) generated at Biogenidec.

### Immunoprecipitation

HEK 293 cells were transfected with combinations of DR6 FL (N-terminal Myc tag), DR6-DN, p75^NTR^, or control cDNA using Fugene HD (Roche). The cells were harvested after 48 h and lysed for 30 min at 4 °C in the lysis buffer described above. After centrifugation at 375 r.c.f. for 15 min, the supernatants were incubated with Protein A/G-Sepharose beads (Santa Cruz Biotechnology, Inc., Dallas, TX, USA) at 4 °C for 1 h and then incubated at 4 °C for 1 h with 4 *μ*g/ml of anti-Myc antibody plus Protein A/G-Sepharose beads. The beads were washed three times with lysis buffer and boiled for 5–10 min in Laemmli sample buffer. Samples were subjected to western blot analysis with anti-Myc (9E10), anti-DR6 (Santa Cruz Biotechnology, Inc.), or anti-p75^NTR^ (Promega, Madison, WI, USA) antibodies.

For immunoprecipitation from human fetal spinal cord tissue, 0.5 mg of spinal cord lysate (BioChain, Newark, CA, USA) was incubated with 4 *μ*g/ml of anti-DR6 antibody (Santa Cruz Biotechnology, Inc.) and analyzed by western blot as described above.

### AP-binding assay

A total of 90% confluent HEK 293 cells were transfected with p75^NTR^ expression plasmid using Fugene HD (Roche). After 48 h, the transfected cells were washed with HBH (Hanks' balance salt buffer supplemented with 1 mg/ml bovine serum albumin, 20 mM HEPES, pH 7.0), incubated for 1.5 h at 23 °C with 4 *μ*g/ml AP-DR6 in HBH, and processed as described.^12^ Bound AP-DR6 was detected by incubation with NBT/BCIP (Roche).

### Europium ELISA

96F MAXISORP black plates (NUNC 437111; Fisher Scientific) were coated with 50 *μ*l/well of 10 *μ*g/ml human DR6 (huDR6) ectodomain in PBS buffer (pH 7.2) and incubated at 37 °C for 1 h. After washing four times with PBS containing 0.05% Tween-20 buffer (PBST), the plates were blocked with 300 *μ*l/well blocking buffer (Hank's buffered salt solution, pH 7.0, 25 mM HEPES, 1% bovine serum albumin, 0.1% ovalbumin, 0.1% non-fat dry milk, 0.001% NaN_3_) for 1 h at room temperature. After washing with PBST, Europium-labeled human p75^NTR^-Fc was added to the plates at a fixed concentration of 1 nM in Perkin Elmer assay buffer. Each sample was run in duplicate with and without competition using 0–400 nM of huDR6 CRD 3-4, huDR6 CRD 1-4, and huDR6 ectodomain. The huDR6 CRD 3-4 and huDR6 CRD 1-4 constructs consisted of residues 132–213 and 42–213 of the full-length human DR6 protein, respectively, and a C-terminal 12-His tag. The huDR6 ectodomain consisted of residues 1–349 of the full-length human DR6 protein. The plates were incubated at room temperature on a plate shaker for 1 h in the dark and washed eight times with PBST. 100 *μ*l/well of enhancement solution (Perkin Elmer) was added and shaken at room temperature for 1 h in the dark. Signal was detected on Victor3 1420 Multilabel Counter fluorometer (Wallac, Perkin Elmer).

### Epitope mapping ELISA

Cell lysates from HEK 293 cells transfected with individual DR6 constructs were processed as described above (immunoprecipitation), and incubated with anti-Myc antibody (Abcam, Cambridge, MA, USA)-coated ELISA plate at room temperature for 2 h. The plate was washed, then incubated with 5D10 (2 *μ*g/ml) at room temperature for 2 h, followed by horseradish peroxidase-anti-mouse IgG (Jackson Immunoresearch, West Grove, PA, USA) at room temperature for 1 h. The signal was detected by 3,3′,5,5′-Tetramethylbenzidine (Thermo Scientific, Waltham, MA, USA) and read at 450 nm.

### Immunohistochemistry and ICC

Tissue sections from adult mouse brain and rat spinal cord were first permeabilized with PBS containing 1% Triton X-100 (Sigma) for 30 min followed by incubation in blocking solution (PBS containing 0.1% Triton X-100 and 10% normal goat serum) for 1 h at room temperature. Cultured neurons were fixed with 4% paraformaldehyde for 30 min, washed, followed by incubation in blocking solution for 30 min at room temperature. For primary antibody labelling, sections or cultures were then incubated in blocking medium containing rabbit anti-DR6 (sc-13106, 1:200, Santa Cruz Biotechnology, Inc.), mouse anti-neuronal *β*III-tubulin (MMS-435P, 1:500, Covance, Denver, PA, USA), and rabbit anti-cleaved casp3 (1:200, Cell signaling) antibodies at 4 °C overnight. After three PBS rinses, sections or cultures were incubated in 5% normal goat serum-PBS containing Alexa 594 anti-rabbit (1:500) or Alexa 488 anti-mouse (1:500) IgG at room temperature for 1 h. The slides were washed and mounted with ProLong antifade with DAPI (4′,6′-diamidino-2-phenylindole dihydrochloride) reagent (Life Technologies) for fluorescence microscopy analysis. DR6 expression in AD and age-matched normal brains (purchased from BioChain) was detected using anti-DR6 antibody (6A12) generated at Biogenidec.

### Generation of DR6-deficient mice

DR6-deficient mice were generated at TaconicArtemis. Briefly, exons 2-3 of DR6 locus were replaced by an Flp-recombination-site-flanked, positive selection cassette. The targeting vector was generated using BAC clones from the C67BL/6J RPCI-23 BAC library and transfected into C57BL/6N Tac ES cell line. Homologous recombinant clones were isolated and used to generate chimeric animals by injection into C57BL/6 blastocysts. Replacing exons 2-3 by the positive selection cassette resulted in loss of expression of the DR6 gene by preventing transcription of exons 2-6. Heterozygous mice were interbred to generate DR6-deficient mice. The genotype of the DR6-locus was determined by PCR using tail DNA. p75^NTR^ null mice were purchased from Jackson Laboratory (Bar Harbor, ME, USA).

### Quantitation of neuronal death

Cultured neurons (WT or DR6 null or p75^NTR^ null) were exposed to A*β*42 for 24 h, with no addition or in the presence of anti-DR6 antibody (5D10, 10–30 *μ*g/ml) or isotype control antibody (MOPC21). Stained neurons were visualized under a fluorescence microscope. Dead neurons were defined as those with shrunken cell bodies, retracted neurites, nuclear DNA condensation, and fragmentation. Apoptotic neuron was quantified with the TUNEL/DAPI system by dividing the number of nuclei exhibiting TUNEL or condensation/fragmentation by the total number of nuclei as identified by DAPI. At least 10 fields were counted for each experimental condition, and each study was repeated two to four times. Aged cortical neurons were within 5–10 divisions.

### Statistical Methods

GraphPad Prism software (GraphPad Software, Inc., La Jolla, CA, USA) was used for statistical analysis. Data are shown as mean and standard error of the mean (S.E.M.). In all studies, comparison of mean values was conducted using unpaired Student's *t*-tests or one-way analysis of variance followed by Tukey *post hoc* test. Statistical significance was determined at the 5% level (*P*<0.05). For all figures, *P<0.05, **P<0.01, ***P<0.001, and ****P<0.0001.

## Abbreviations

A*β*: *β*-amyloid
AD: Alzheimer's disease
AP-DR6: alkaline phosphatase–DR6 fusion protein
casp3: caspase 3
CNS: central nervous system
CRD: cysteine-rich domain
DAPI: 4′ 6′-diamidino-2-phenylindole dihydrochloride
DR6: death receptor 6
DR6-DN: DR6 dominant negative
DR6 FL: full-length DR6
ICC: immunocytochemistry
LINGO-1: Leucine-rich repeat and Ig domain containing NogoR interacting protein 1
N-APP: N-terminal *β*-amyloid precursor protein
NogoR: Nogo receptor
NT: neurotrophin
p75^NTR^: p75 neurotrophin receptor
PBS: phosphate-buffered saline
RT-PCR: real-time PCR
TNFR: Tumor necrosis factor receptor
TRAILR: TNF-related apoptosis-inducing ligand receptor
TrkA: tropomyosin receptor kinase
